# Supplementary material for: Assessing the Role of AtGRP7 Arginine 141, a Target of Dimethylation by PRMT5, in Flowering Time Control and Stress Response
Source: Plants (Basel). 2024 Oct 3;13(19):2771. doi: 10.3390/plants13192771 (PMC11478431; doi:10.3390/plants13192771)
Supplement: Supplementary file 1 [file plants-13-02771-s001.zip › plants-3238706-supplementary.pdf]

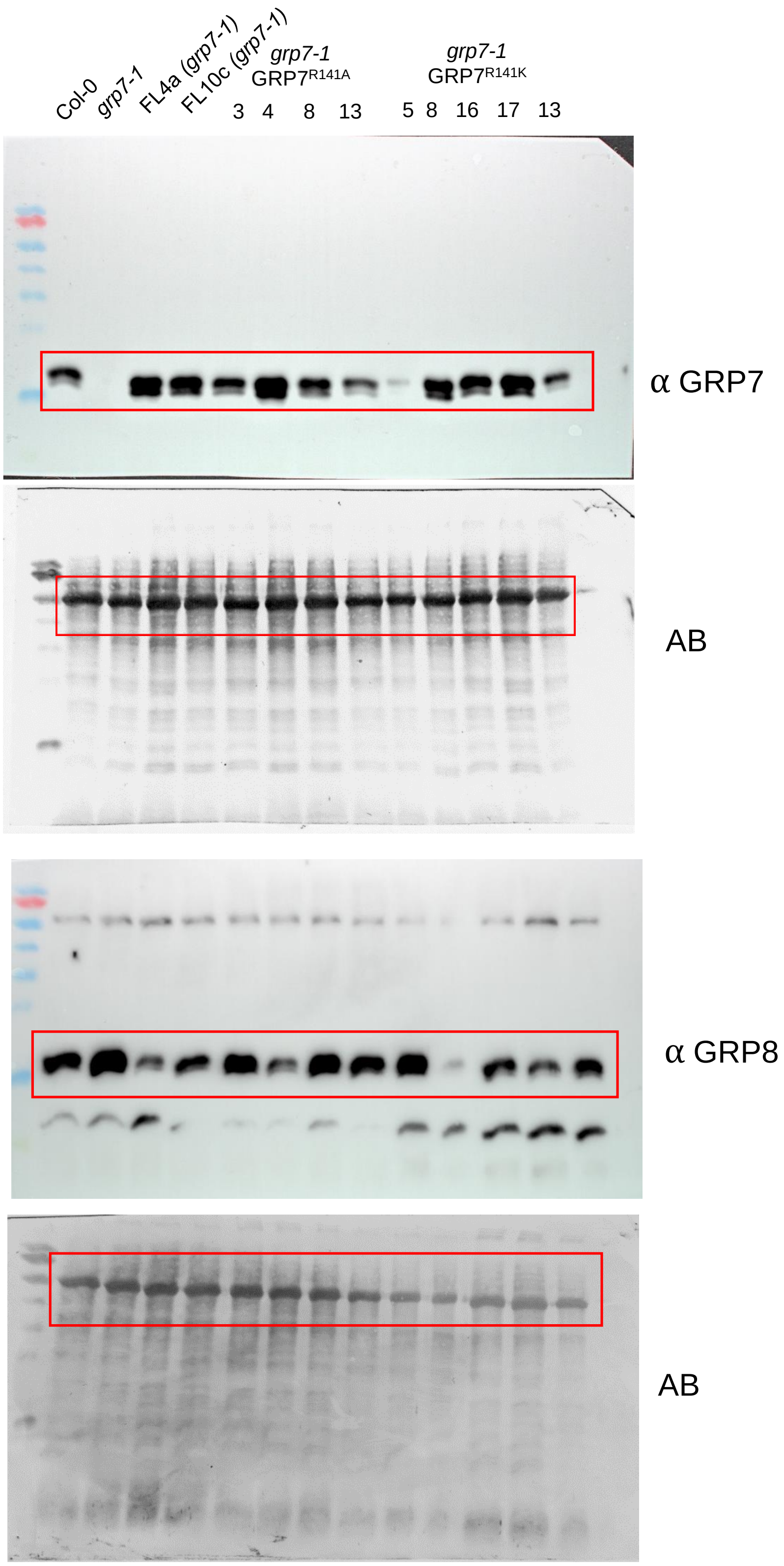

Supplementary Figure S1. Uncropped blots corresponding to Fig. 1. Cropped areas highligted with red rectangle.

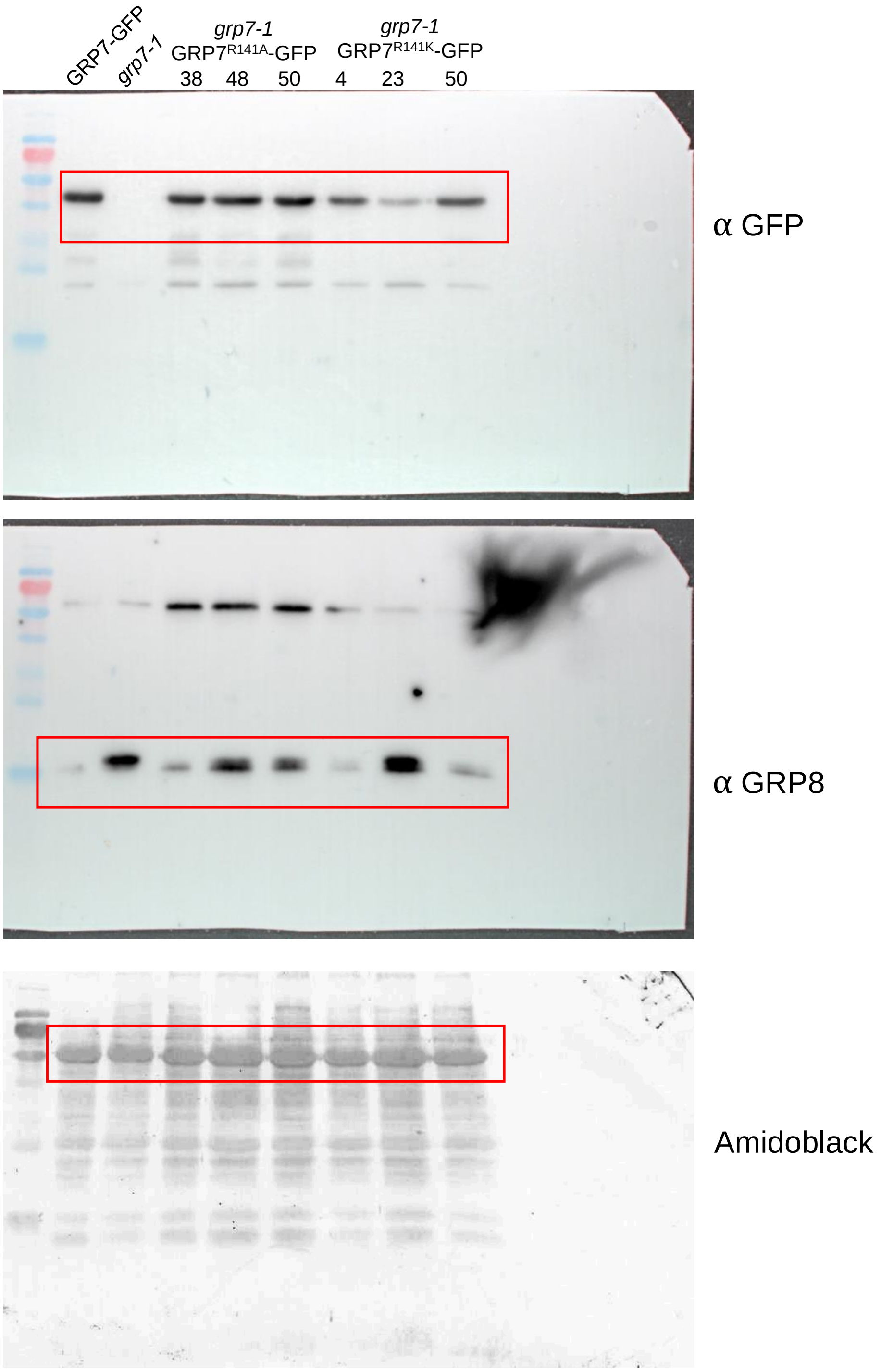

Supplementary Figure S2. Uncropped blots corresponding to Fig. 2. Cropped areas highligted with red rectangle.

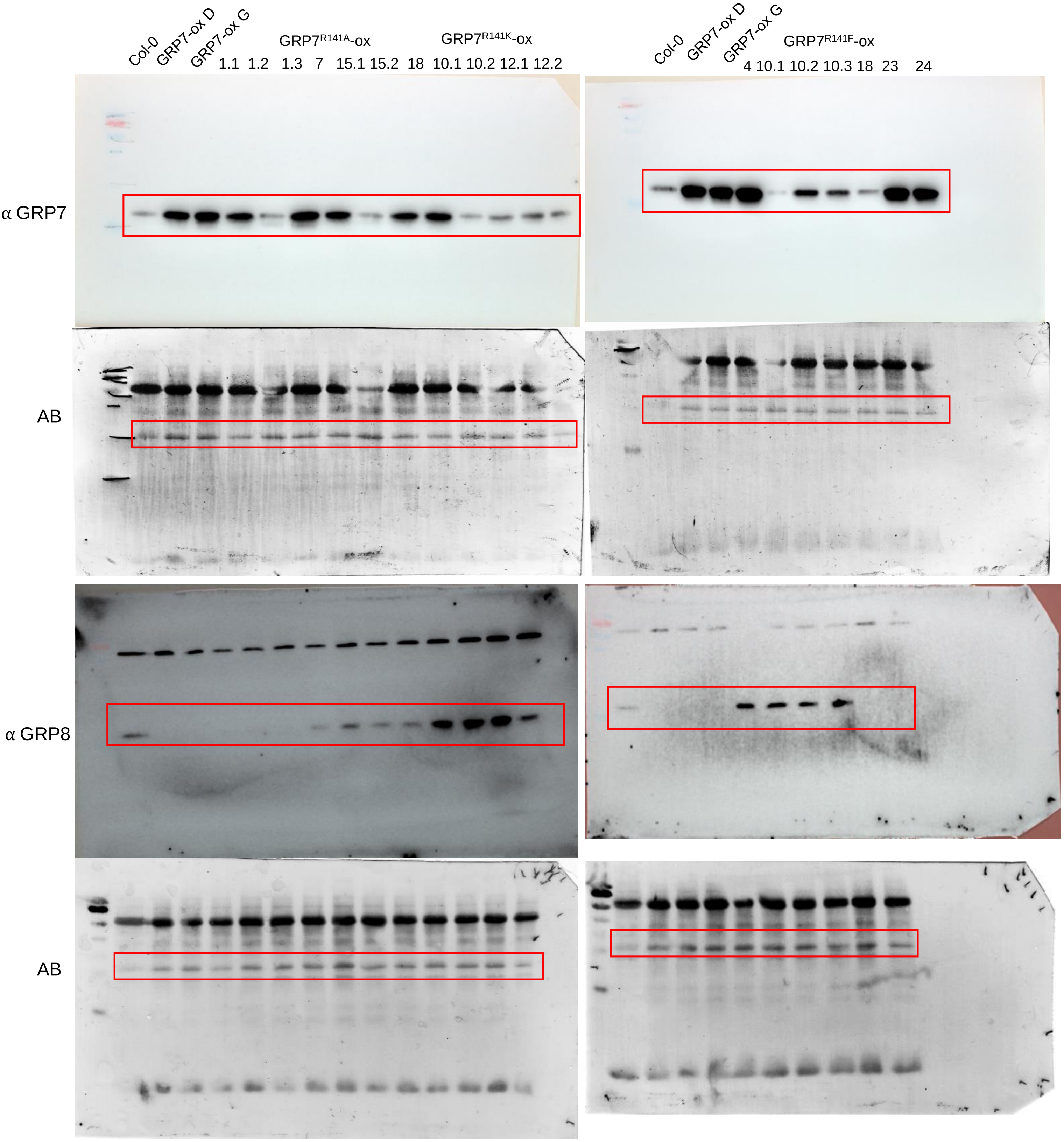

Supplementary Figure S3. Uncropped blots corresponding to Fig. 3. Cropped areas highligted with red rectangle.

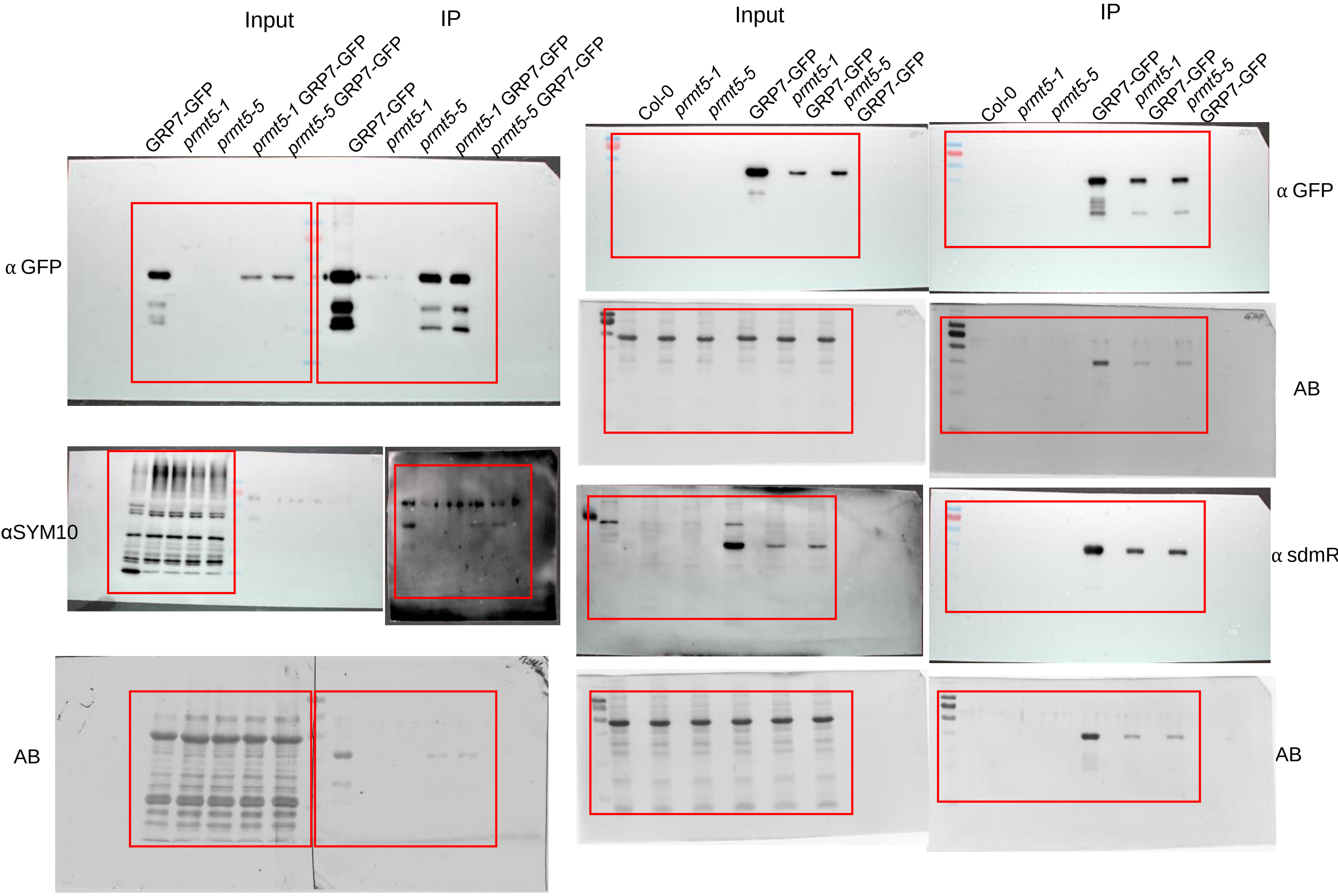

Supplementary Figure S4. Uncropped blots corresponding to Fig. 5. Cropped areas highligted with red rectangle.

Supplementary Table S1. Oligonucleotides used in this study

**Oligonucleotide**	**AGI**	**Symbol**	**Sequence**	**Properties**
prmt5-5 fwd	AT4G31120	PRMT5	ATAGCCAGCGAATCCTATTATG	Genotyping prmt5-5, KpnI cuts wt, not prmt5-5
prmt5-5 rev	AT4G31120	PRMT5	CTTTCTCCTGAGTGTCTTGATG	Genotyping prmt5-5, KpnI cuts wt, not prmt5-5
cx166	AT4G31120	PRMT5	TCTTGTGACAAAAATACAGCACAA	Genotyping prmt5-1, SALK_065814
cx167	AT4G31120	PRMT5	CATCCATTTGGCAGGTTAAGGC	Genotyping prmt5-1, SALK_065814
AtGRP7F(1)TDS	AT2G21660	AtGRP7	CGTGATATGTCCCCAACCACTACGA	Genotyping grp7-1, SALK_039556
Agrp54	AT2G21660	AtGRP7	AGATGAACGTACCGATTTGGGA	Genotyping grp7-1, SALK_039556
LBb1.3			ATTTTGCCGATTTCGGAAC	left border primer, SALK T-DNA lines
grp7RF +	AT2G21660	AtGRP7	GGTTACTCCTCATTCGGTGGTGGTGGCGGAAGCTAC	site directed mutagenesis AtGRP7R141A -> R141F
grp7RF -	AT2G21660	AtGRP7	GTAGCTTCCGCCACCACCACCGAATGAGGAGTAACCG	site directed mutagenesis AtGRP7R141A -> R141F
